# Paranoia

**Published:** 1898-10

**Authors:** Ross George Loop

**Affiliations:** Buffalo, N. Y., Interne Erie County Hospital


### PARANOIA.

By ROSS GEORGE LOOP, M. D , Buffalo, N. Y.,
Interne Erie County Hospital.

OF THE various forms of mental derangement, due to disease of the brain, there is perhaps none more interesting than that variously known as monomania, primary delusional insanity or paranoia. The term paranoia is derived from two Greek words *Ταρά* and *Γόος*, meaning beside the mind, and is defined as a mental disease, characterised by systematised delusions, in which heredity usually plays an important rôle. The main fundamental characteristic of the disease is a delusion, which becomes part of the belief of the individual and which he considers himself able to explain and defend.

This disease is more rarely met with than is either mania or melancholia, and differs quite radically from them in most of its manifestations. The systematised delusions, which are the essential features of the condition, may be either expansive or hypochondriacal in character, or both may be present in the same patient at different times. As these delusions of persecution and grandeur form neither a part of, nor of necessity follow other abnormal mental conditions, the term primary delusional insanity perhaps best comprehends the mental state which it is intended to designate. The intellect is rarely much involved and this class of patients were formerly included under the head of the partially insane. They are dangerous to be at large, owing to their peculiar delusions and apparent rationality. "They have in themselves a law sufficient unto themselves."

*Etiology.* — Paranoia is a neurosis in which the element of heredity is very marked. A predisposition to the disease is usually born with the individual. There is often a history of insanity or at least of a nervous temperament in one or both parents. Maternal impressions, malformations of the skull, injuries to the skull, infantile convulsions or delirium, diseases of childhood, such as meningitis, over-study in children all predispose to the acquired form, which may manifest itself in early life or adolescence. It is also often associated with the period of puberty, adolescence and the climacteric, while masturbation and systemic diseases predispose to the development of the condition. It occurs more frequently in females, celibates and especially those born out of wedlock. It comes especially under the class of degenerative diseases. The stigmata of degeneration, such as asymmetry of the skull, irregularities of the jaw, palate, nose, ears and eyes, are quite frequently but not invariably present.

Paranoia exists in two forms, viz.: the primary or Wahnsinn and the secondary or Verrücktheit and several sub-divisions, such as the ambitious, religious, querulent, erotic and the like are made.

*Morbid Anatomy.*—The pathological anatomy is not well understood. There seems to exist a morbid sensitiveness of the cerebrospinal system.

*Symptomatology.*—I. Wahnsinn—This form of paranoia comes on quite suddenly, the delusions and hallucinations being rapidly combined and associated with strong effects. There is usually considerable mental excitement and delusions of depression are interspersed with those of exaltation, thus simulating mania circulaire, these periods not being so definite, however, as in the latter-named disease. The depressing delusions resemble also melancholia, but the patient does not on their account suffer mentally; he never despairs; he anticipates and considers escape from the persecutions as possible, if only the right methods are employed.

The more violent and characteristic symptoms are usually preceded by a longer or shorter period of insomnia and irritability, during which the patient may draw unpleasant conclusions from ordinary perceptions. Following this prodromal stage, delusions of persecution become dominant. He grows suspicious of his friends and relatives; while outwardly friendly and attentive, they are really plotting to do him injury; he hears people making remarks about him on the street; he is suffering unjustly; he is always right and no one can explain any falsely-interpreted remarks or noises to him. There may be long periods of terror and systematised delusions and hallucinations with now and then a period of interruption, not, however, due to exhaustion, as in mania. The patient sleeps well, and is well nourished and may be quite rational, except in regard to his delusions.

Those cases characterised by delusions of grandeur are chiefly religious. The patient is inspired. He sees heavenly visions and hears revelations. He may have exalted delusions regarding his physical development and be fond of appearing in public in gaudy attire.

The prognosis of Wahnsinn is unfavorable. The symptoms may subside, but the improvement is frequently of only temporary duration and often terminates in confusion or dementia.

*Treatment.*—In this form of paranoia, sedatives such as the bromides—sulphonal or trional—and nonstimulating tonics are indicated.

The prolonged use of iron is highly recommended. A well-regulated diet and exercise are also essential. Isolation is best when illusions exist and company where hallucinations are prominent. In prolonged cases animal extracts are said to be very valuable.

II. Verrücktheit.—In this form of paranoia, delusions of persecution and grandeur, usually with hallucinations, are carefully and slowly combined into a progressive delusional system. There is almost always an hereditary taint and the stigmata of degeneration are frequently noticeable. It usually runs an unfavorable course. There is a permanent and profound change in personality, but consciousness remains unaffected. In childhood there is manifested an unnatural love of solitude and interest in the subjective rather than the objective. At puberty this tendency to seclusion continues and distrust and suspicion develop. "Long before recognised as actual lunatics they are styled 'cranks.'"

Delusions of exaltation and depression are noticed, although concepts are rationally combined in so far as they are not opposed to the dominant delusions. The delusions and hallucinations have not yet obtained convincing reality to the patient, who tries to overcome them by reasoning, but with only temporary success, thus showing the loss of the power of criticism. Real misfortunes do not affect him and he attributes them to their proper causes. Retrospection and combination of past events with fresh impressions is noticed, thus forming a chain of occurrences all going to prove to the satisfaction of the patient the persecutions to which he is subjected. Auditory hallucinations also develop. The patient hears depreciating remarks about himself; he is mocked and insulted by the people on the street. Generally, he does not know his enemies and makes no effort to find them out, thus showing his failing judgment. He is threatened on every hand and at last attempts to escape by going to some remote part of the country. This usually gives but temporary relief, as his enemies soon get on his track again and renew their plots against him. Thus he may travel from place to place, hoping to evade his tormenters, and at last, despairing of this, he may become less reticent and timid and may even speak to his persecutors about their inimical conduct. He may then become more secluded than ever. He locks his doors and windows; stops the keyholes; cooks his own food, and, in short, shuts himself away from the world as much as possible. But escape seems impossible—his enemies read his thoughts and he hears them spoken almost before they are formed. At length he appeals to the civil authorities for protection, and this failing, he naturally concludes that they are in league with his persecutors.

Hallucinations regarding their physical condition are common. The patient may declare that his brain or spinal cord have been torn out; he has pain in the viscera, and the like. There are also hallucinations of the special senses. The air is full of disagreeable odors and the food tastes like excreta. Hallucinations of vision are less common. Delusions of grandeur may develop primarily or as a result of those of persecution. He may conclude that the general attention and observation to which he imagines himself subjected is due to his greatness. He now experiences new sensations of magnetic currents in his body. He awakens to a new life and may suddenly announce that he is a great ruler, a prophet, or a Son of God, showing the frequent religious basis of Verrücktheit.

In the so-called ambitious form, delusions of grandeur are constantly present. It is to this class that the political reformers, Nihilists and Anarchists, such as Guiteau and Prendergast, belong, while those who direct their attentions toward people in a higher plane of society, *e. g.*, where a laboringman falls in love with a queen or princess, belong to the erotic form. Later the patient may become suicidal and dangerous, attacking his supposed enemies. These patients are frequently very bright. They often coin new words to express their ideas, and many remarkable discoveries and inventions have been made by them. One of the best German-English lexicons was compiled by a paranoiac while confined. Hallucinations are rare in persons not previously bright mentally.

Verrücktheit is common, especially in females, and begins usually at puberty, although it may develop at any age. It very rarely begins in old age. The disease is usually preceded by a period of depression and irritability, but may come on suddenly with violence.

It progresses by fits and starts, rather than by gradual advancement, and during its course there are marked remissions. The prognosis is unfavorable. It runs a chronic course and may terminate in incomplete recovery, the chronic state, dementia or death. Recovery is rare, it being generally incurable, but patients may become at least harmless. They are dangerous to society during the disease, but after the development of confusion they need not be confined. Much can be accomplished when peculiarities are manifested in childhood by training the child properly, teaching him to bridle his imagination and encourage healthful sports and exercise—arousing his interests in the objective and drawing his mind from the subjective.

Many of the foregoing symptoms are illustrated by the following cases, two of which have never been reported:

CASE I.—Samuel L. Age, 33. Born in United States. Family history negative as far as could be learned. Physical condition good. He has a scaphocephalic head, high cheek-bones, narrow forehead, and slightly protruding chin. He rarely looks at the person he addresses, but gazes fixedly in the distance. His voice is low and he speaks slowly and with an air of conviction. He has the delusion that he is "mesmerised" by a band of electricians and millionaires. They first gained control over him, and have since held it, by compelling him to masturbate whenever they see that their power over him is growing weaker. He has been in their power since he was fourteen years of age and has traveled from one end of the country to the other in vain hope of evading their influences. Says that he is their agent in a gigantic plot to overthrow the United States, that he is unconscious when he performs their errands, but without him their plans would fail. He does not know these persecutors, nor where they are, but says he would kill them if he had the chance. They exercise their complete power over him, not alone to aid their own plans, but even in trifling matters he is not allowed his own way, he must walk on certain streets, eat at certain times and places, and the like, as directed by them. He says his brain and spinal cord have been ruined by the constant action of electricity. He has worked at various kinds of labor all over the United States, rarely staying more than one week in a place because "things get too damned hot" for him. He would like either to commit suicide or else inform the United States government of its danger, but his controllers will not allow him freedom to do either. They make his food taste like urine and feces; his sleep is disturbed by voices, and during waking hours he is never at ease.

After daily visits to the university of Buffalo dispensary for nearly two weeks, asking for medical treatment and protection, he suddenly ceased to appear, and has probably again taken up his weary and futile journey for escape.

His three dominant delusions, viz.: That he is persecuted by unknown men, that his brain and spinal cord are destroyed, and that he is the central and all essential figure in this great plot against the government, are very typical of this disease.

CASE II.—Through the courtesy of Dr. Crego and the officers of the Buffalo State Hospital I am permitted to cite the following case: B.———, aged 33 years, was admitted in November, 1892. Occupation, none. Family history not known.

Habits good. Has always been an erratic, queer individual, leading an unsettled life, wandering about the country doing peculiar things. Studied medicine for a short time and was expelled from college for misconduct. Has squandered a large sum of money, but labors under the delusion that a clergyman defrauded him and has threatened to kill him if the money is not refunded. For a time after admission talked incessantly, was irritable and suspicious, refused medicine and ate sparingly. He is now rather nervous in his actions, has some vaso-motor disturbance, eyes bright and restless, voice low and tremulous, is negligent in dress and personal appearance. Has prominent cheek-bones, narrow forehead, protruding lower jaw, with eyes set deeply in orbits. Says he has made exhaustive studies in drugs, by noting their action on himself, so that he can tell the exact quantity, quality and constituents of all medicines taken, that his brain is more highly developed than that of any living man, that his perceptions, which are located over his eye-brows, are so large, that notwithstanding the fact that doctors have tried to ruin his brain by thousands of drugs, they (the perceptions) still overlap each other, the perception of individuality being especially developed. He challenges any one to box or lift weights with him, or he offers to meet any one in an argument on any subject and show his superiority, without previous preparation. The systematised delusions of persecution and exaltation are strikingly illustrated by his statement: "They are trying to ruin the brain of the greatest living thinker." He is not despondent, and says that his persecution and confinement, like all martyrs, is because his ideas are in advance of his time.

CASE III.—T. ———, aged 38 years. Born in Germany. Parents dead. Father was eccentric, lead a secluded life and was brutal to his family. Personal: From boyhood up he has been egotistical, selfish and suspicious and is constantly in trouble with those with whom he is associated. Says that certain people are talking about him, making his employer and fellow-workmen dislike him, that he understands his trade better than any man in the world. His married life was unhappy. Suspicious of his own relatives, he refused them admission to his house and threatened to kill his wife if she visited her parents, and finally drove her from the house at night, and soon after left town. It was found that he had squandered a large sum of money recklessly and purposely. Since then he has traveled from one place to another, doing queer things, only sojourning a short time in one place, for his persecutors follow him, defaming his character and disturbing him constantly.

